# Tailoring cardiovascular risk assessment and prevention for women: One size does not fit all

**DOI:** 10.21542/gcsp.2017.1

**Published:** 2017-03-31

**Authors:** Nanette Wenger

**Affiliations:** Professor of Medicine (Cardiology) Emeritus, Emory University School of Medicine Consultant, Emory Heart and Vascular Center, Atlanta, USA; Founding Consultant, Emory Women’s Heart Center

## Introduction

**Figure 1.**
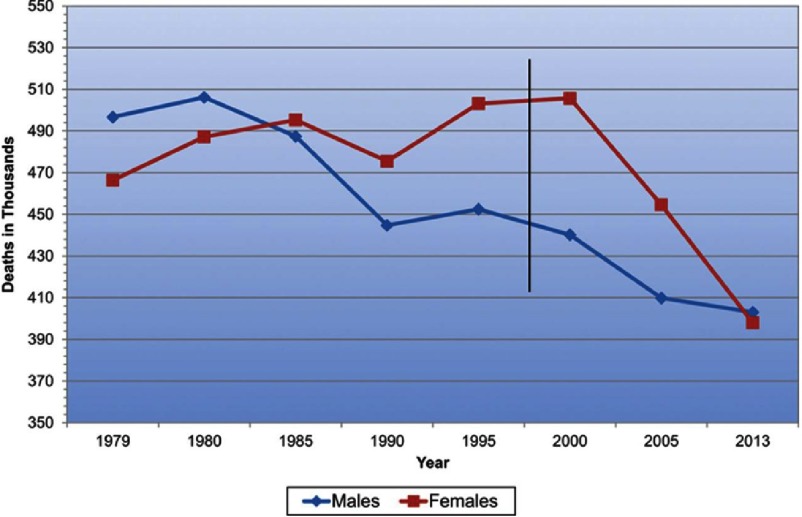
CVD mortality trends for males and femaies (US, 1979–2013). Reproduced with permission from [1].

For many years, cardiovascular disease was considered predominantly a disease of men, despite the fact that more U.S. women than men died annually from cardiovascular illness.^1^ Because of this misperception of their cardiovascular risk, for many years women were underdiagnosed and undertreated, with consequent increases in cardiovascular morbidity, mortality, and disability. With recent appreciation of this historical gender disparity, clinical and epidemiological research studies have identified cardiovascular features specific to women, resulting in an improved spectrum of care. Although since 1984 more U.S. women than men died annually from cardiovascular disease, beginning in the year 2000 there was a sharp decline in cardiovascular mortality for women, indeed, more precipitous than that for men. Half of this favorable effect is considered due to improved preventive strategies and the remainder to improved management of recognized cardiovascular disease. In 2013, for the first time, more U.S. men than women died of cardiovascular disease and we are delighted to be in second place.^1^ [Figure 1: CVD Mortality Trends for Males and Females (United States 1979–2013).]

## Models for Risk Assessment

### The Framingham Risk Score

The initial assessment model for the estimation of coronary risk was the Framingham Risk Score (FRS), first published in 1998^2^ and revised in 2008. It provides a 10-year estimate of coronary heart disease risk; a problem of this short-term risk estimation is that women traditionally have a lower short-term cardiovascular risk but a higher lifetime risk. Thus, the Framingham Risk Score characteristically underestimates cardiovascular risk for women. As an example, in the MESA Study,^3^ women in the highest quartile of coronary calcium scores were still characterized as at low risk by the Framingham Risk Score.

### The Reynolds Risk Score

The predominant differences between the Framingham Risk Score and the Reynolds Risk Prediction model is the inclusion of high sensitivity C-reactive protein and a family history of myocardial infarction in the Reynolds calculation.^4^ As with the Framingham Risk Score, the Reynolds Risk model fails to incorporate a number of the risk attributes unique to or predominant in women, as will be discussed later.

### 2011 Effectiveness-Based Guideline for the Prevention of CAD in Women Algorithm

This guideline attempted a simplification of the risk assessment for women. Women were classified as at ideal cardiovascular health, at-risk for, or at high risk for cardiovascular disease.^5^ Ideal cardiovascular health was characterized as a total cholesterol of <200 mg/dL (untreated), a blood pressure <120/80 mmHg (untreated), a fasting blood glucose <100 mg/dL (untreated), a body mass index <25 kg/m^2^, abstinence from smoking, physical activity at goal, i.e., ≥150 minutes/week of moderate intensity or ≥75 minutes/week of vigorous activity or a combination, and a heart healthy diet.

Women considered at risk had ≥1 major risk factor; these included cigarette smoking, SBP ≥120 mmHg, DBP ≥80 mmHg or treated hypertension; total cholesterol ≥200 mg/dL, HDL-C ≤50 mg/dL, or on treatment for dyslipidemia; obesity; poor diet; and physical inactivity. Other at-risk characteristics included a family history of premature CAD in a first degree relative, metabolic syndrome, evidence of advanced subclinical atherosclerosis, a poor exercise capacity on treadmill testing and/or abnormal heart rate recovery, the presence of systemic autoimmune collagen-vascular disease (i.e., SLE or RA), and a history of gestational diabetes, preeclampsia or pregnancy-induced hypertension.

A high-risk status was characterized by ≥1 high risk state which included clinically manifest CVD, clinically manifest cerebrovascular disease, clinically manifest peripheral arterial disease, abdominal aortic aneurysm, end-stage or chronic renal disease, diabetes mellitus or a 10-year predicted CVD risk ≥10%.^5^ Importantly, the 2011 Women’s Prevention Guideline antedated the 2013 ACC/AHA Prevention Guidelines.

### ACC/AHA Pooled Cohort Equation

**Figure 2.**
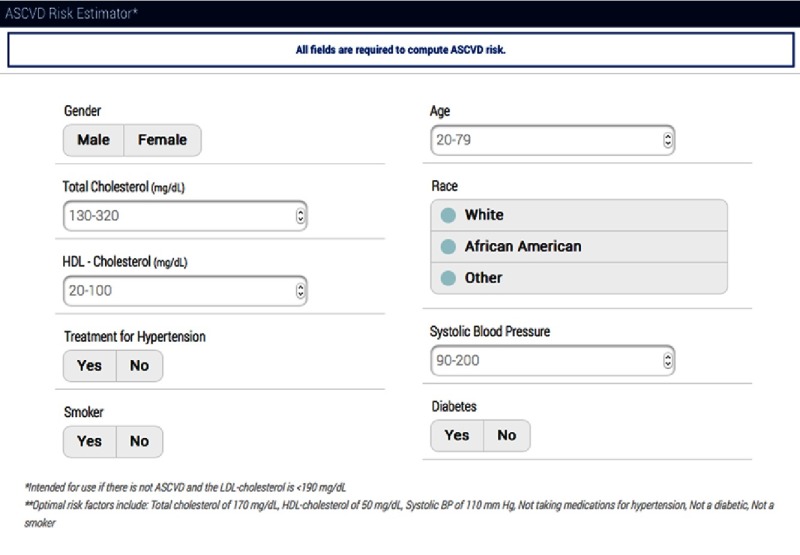
ASCVD Risk Estimator. Source http://tools.cardiosource.org/ASCVD-Risk-Estimator/.

More recently, the American College of Cardiology and American Heart Association provided a new calculator for the estimation of cardiovascular events (http://tools.cardiosource.org/ASCVD-Risk-Estimator/) which is gender-specific and provides specific information for Caucasians and African Americans (Figure 2: ASCVD Risk Estimator). The Pooled Cohort Equation provides both a 10-year atherosclerotic cardiovascular risk and a lifetime risk. The advantage for women is that their higher lifetime cardiovascular risk is included in this assessment the clinician provides to the woman. Its use is delineated in the 2013 ACC/AHA Guideline on Assessment of Cardiovascular Risk.^6^ A concern is the overestimation of cardiovascular risk with advanced age.^7–9^

### Prevention of stroke in women

**Figure 3.**
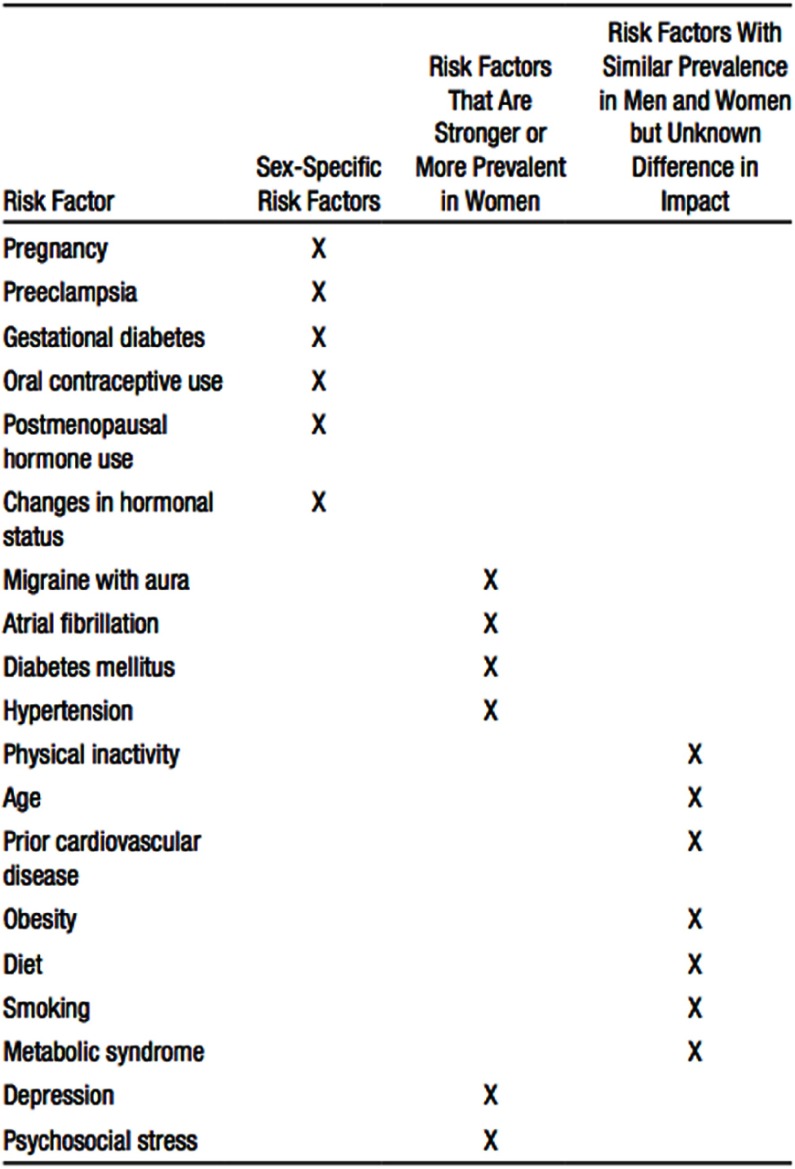
Guideline for the prevention of stroke in women. Reproduced with permission from [10].

A recent statement from the American Heart Association and American Stroke Association defines the risk factors for stroke sex-specific for women and those more prominent in women (Figure 3: Stroke Risk Factors).^10, 11^

### My approach for cardiovascular risk assessment in asymptomatic women

I use as the basis the ACC/AHA Pooled Cohort Equation, but incorporate as well the risk factors unique to or predominant in women (as discussed below). I also consider the sex-specific components of stroke risk. See Figure 3 for further details.

## Evaluation of Symptomatic Women for Ischemic Heart Disease

The AHA recommends a treadmill exercise test as the initial diagnostic test for symptomatic women with an intermediate pre-test likelihood of CAD.^12^ This assumes a functionally capable woman, i.e., an appropriate ability to exercise and an interpretable resting electrocardiogram. For clinicians unsure of a woman’s functional capacity, the 12-item Duke Activity Status Index (DASI) can estimate the metabolic equivalents (METs) associated with activities of daily living, identifying women unable to achieve 5 METs who should be considered for pharmacologic stress testing.^13^ The Duke Treadmill Score (DTS) provides valuable information about functional capacity and prognostic information for the risk stratification of women;^14–16^ a low DTS is associated with <1% annual mortality vs an annual mortality of nearly 5% in women with a high DTS.^14^ Low functional capacity is associated with a higher occurrence of cardiovascular event rates. Importantly, a normal stress ECG has a very high negative predictive value.

Stress echocardiography or SPECT imaging is appropriate for intermediate-risk symptomatic women with a poor functional capacity or an abnormal resting electrocardiogram precluding ST segment interpretation during exercise testing. The addition of imaging to a treadmill test improves the diagnostic accuracy, with echocardiography preferred compared with nuclear imaging because of the absence of radiation exposure. The sensitivity and specificity of ETT for the detection of obstructive coronary disease increases from 31–71% and 66–86% respectively to 80–88% and 81–86% respectively with exercise stress echocardiography compared with ETT.^17–20^ Pharmacologic stress testing has a diagnostic sensitivity of 91% and specificity of 86% in women and also provides information on the extent of ischemic perfusion defects and ventricular function. Stress photon emission tomography (PET) also improves the diagnostic accuracy of detecting obstructive CAD in women with suboptimal stress SPECT imaging or poor windows for stress echocardiography; however, exercise cannot be performed during stress PET testing and pharmacologic therapy is indicated. Stress cardiac magnetic resonance (CMR) imaging has the advantage of avoiding radiation compared with stress PET or SPECT, with increasing data being acquired as to its sensitivity and specificity. Computed coronary tomographic imaging (CCTA) uniquely provides information on the burden of obstructive coronary disease.

## Traditional Risk Factors for Cardiovascular Disease

Traditional risk factors have a differential impact by gender. The best studied of the traditional risk factors are diabetes mellitus, tobacco use, hypertension, lipids and lipoproteins, obesity, and physical inactivity.^21^

### Diabetes mellitus

Women with diabetes have a 3- to 7-fold increased likelihood of developing coronary disease than nondiabetic women versus diabetic men whose risk is 2- to 3-fold compared with nondiabetic men.^22^ Particularly in young and middle-aged women, who generally have a lower occurrence of coronary heart disease than comparably aged men, diabetes is associated with a 4- to 5-fold increased rate of CAD.^23^

Diabetes confers a greater cardiovascular risk for women than men, 19.1% vs 10.1%. Diabetic women have a 40% greater risk of incident coronary disease and a 25% excess in stroke risk. More women than men at the time of presentation of an initial myocardial infarction are diabetic, 25.5% vs 16.2%. The correlation of cardiovascular mortality is greater in diabetic women than diabetic men. It remains uncertain whether this is related to an increase in adiposity, excess abdominal adiposity, or insulin resistance, among others. Nonetheless, diabetic women have a far more adverse cardiovascular risk profile than do diabetic men. Additionally, diabetic women have less appropriate treatment and control of cardiovascular risk factors than their male peers. Diabetic women were the sole group without mortality improvement between 1971-75 and 1982-84 in the U.S. Mortality in these temporal groups decreased in men with and without diabetes and women without diabetes (13%, 36%, 21% respectively). However, mortality increased among diabetic women 23%.^23–27^

### Tobacco use

16.7% of women in the U.S. smoke and younger women are more likely than younger men to initiate smoking behavior. There is a 25% increase in cardiovascular risk for women as compared with men smokers,^28^ with cigarette smoking tripling the risk for women for myocardial infarction. Tobacco use selectively disadvantages women who incur a higher risk of myocardial infarction compared with men.^29^ Cardiovascular surgeons often report that they rarely perform coronary artery bypass graft surgery in women younger than 50 years of age, absent a history of tobacco use. Smoking cessation is the most cost effective cardiovascular risk modification strategy in the U.S.

### Hypertension

Hypertension is the leading cause of cardiovascular mortality worldwide, with an increased population-adjusted cardiovascular mortality for women compared with men, 29.0% vs 14.9%.

Hypertension is more prevalent in U.S. men than women until about age 45, with a higher prevalence in women after age 55-65.^1, 30^ Multiple studies show that women with hypertension are less likely to be treated to goal.^31^

There is an impressive correlation with body mass index and elevated systolic blood pressure in women. Notably 80% of U.S. women aged 75 and older have hypertension, but this increase in blood pressure with age is not present in non-industrialized societies, identifying the likelihood of substantial societal contributions to hypertension. In the U.S. 20% of elderly women have adequate blood pressure control in contrast to the 41% of elderly men.^24, 32–35^

### Lipids and lipoproteins

After menopause, lipoprotein levels change, with rising triglyceride and low-density lipoprotein cholesterol (LDL-C) levels and a decrease in high-density lipoprotein cholesterol (HDL-C) levels. The degree to which these are impacted hormonal changes and lifestyle changes is uncertain, as menopausal women often have an increase in body mass index and a decrease in physical activity. Nonetheless, the unfavorable lipid profile in menopausal women is associated with a cardiovascular risk approaching that for men. Despite comparable lipid lowering benefit, women are less likely to be treated with statins than are men after myocardial infarction.^36–39^

Elevated cholesterol imparts the highest population-adjusted cardiovascular risk for women, 47%, with similar statin benefit shown for women and men. The 2013 ACC/AHA Guidelines recommended significant changes in the management of dyslipidemia. Risk was assessed based on the new Pooled Cohort Risk Equations and lifestyle guidelines (dietary and physical activity) were recommended as initial management for LDL-C lowering. Fixed-dose statin therapy was recommended for both women and men based on risk categorization, with no target LDL-C levels. Moderate-dose statins were recommended for both women and men older than 75 years and non-statin therapies were not recommended. The latter issue antedated the clinical trial data showing benefit from ezetimibe added to statin therapy, and from PCS K9 inhibitors; both therapies now must be considered in the management of elevated LDL-C in women.

This composite will likely increase statin use in women, probably decrease inappropriate use, decrease non-statin use, and certainly lessen laboratory testing.^6, 24, 32, 40, 41^

### Obesity

Two out of every three U.S. women are obese or overweight based on 2010 data. Obesity is associated with hypertension, dyslipidemia, physical inactivity, and insulin resistance. Obesity increases coronary risk more for women than for men, 64% vs 46%. Of interest is that obesity is double in women compared with men in low-middle income nations, whereas obesity is equivalent in women and men in high-income nations.^24, 42, 43^

### Physical inactivity

32% of U.S. adults are physically inactive, 33.2% of women compared with 29.9% of men. Physical inactivity is the most prevalent risk factor for U.S. women, with one quarter of U.S. women reporting no regular physical activity, and 34% reporting less than the recommended amount of daily activity. In the INTERHEART study, the protective effects of exercise appeared greater for women than for men.^24^ Beneficial physical activity data specific to women derive from the Nurses’ Health Study, where there was a decreased development of type 2 diabetes among women who exercised regularly; as well, among diabetic women in the Nurses’ Health Study, physical activity decreased the risk of cardiovascular events. For secondary prevention, exercise-based cardiac rehabilitation is a Class 1A recommendation in all U.S. clinical practice guidelines; despite this, women are 55% less likely than men to participate.^24, 32, 40, 44, 45^

## Cardiovascular Risk Factors Specific to or Predominant in Women

### Systemic autoimmune disorders

Systemic autoimmune disorders are highly prevalent in women, including systemic lupus erythematosus, rheumatoid arthritis, and psoriasis, among others.

Although the mechanism is not well understood, a robust literature demonstrates that systemic lupus erythematosus and rheumatoid arthritis are associated with an increased risk of coronary disease, independent of traditional cardiovascular risk factors,^46–49^ with systemic autoimmune disorders predominating in women. Accelerated atherosclerosis is a well-recognized finding in this population.^46, 50, 51^ Patients with SLE have premature cardiovascular mortality, with a mean age of 52 for myocardial infarction in one study.^52^ Cardiovascular disease is the leading cause of morbidity and mortality in patients with SLE, and patients with RA also have an increased risk of ischemic heart disease; they have a doubled risk of developing heart failure and a 1.5- 2-fold risk of CAD. Psoriatic arthritis also increases CVD event risk but not CVD mortality.^53, 54^

Systemic autoimmune collagen vascular disorders pose an increased risk for both coronary heart disease and cerebrovascular accident. Indeed, coronary disease is the leading cause of morbidity and mortality in patients with systemic lupus erythematosus. There is a 2- to 3-fold increase in myocardial infarction and cardiovascular mortality in women with rheumatoid arthritis. Thus this warrants screening for cardiovascular risk in these women and institution of appropriate preventive interventions.^5, 53, 55^

#### Hypertensive and diabetic complications of pregnancy

Pregnancy complications, including preeclampsia, gestational diabetes, pregnancy-induced hypertension, pre-term delivery, and small for gestational age weight are all early indicators of an increase in cardiovascular risk.^56–58^ It has been said that pregnancy is the first stress test a woman undergoes, in that the cardiovascular and metabolic stresses of pregnancy have the potential for the early prediction of cardiovascular risk. It is unlikely that the complications *per se* impose risk, but likely that there are shared risk factors between preeclampsia and cardiovascular disease. With preeclampsia there is a 3- to 6-fold increase of subsequent hypertension and a doubled risk of subsequent ischemic heart disease^59–62^ and stroke. Although the preeclampsia subsides with delivery, there remains residual endothelial dysfunction and there is a prominent association of these complications with an increase in coronary artery calcium, evidence of coronary atherosclerosis. A 2017 report of the US Preventive Services Task Force recommended that pregnant women have their blood pressure checked at each prenatal visit to screen for preeclampsia. Gestational diabetes imposes a 7-fold increased risk of subsequently developing type 2 diabetes^5, 61–64^ and an increased future risk of cardiovascular disease.^59, 64, 65^ Therefore, a detailed pregnancy history is an integral component of risk assessment for women.

#### Oral contraceptive therapy

Oral contraceptives increase blood pressure in most women, but rarely are associated with malignant hypertension. Different oral contraceptive formulations have variable effects on blood pressure. The most adverse effect is seen in association with cigarette smoking. Oral contraceptives with low-dose estrogen increased the risk of hypertension in the Nurses’ Health Study,^30^ but oral contraceptive discontinuation is typically associated with a return to baseline blood pressure values within a few months.^66^

Oral contraception does not impose an increase in cardiovascular risk among healthy women with no risk factors. However, smoking and oral contraception are associated with a 7-fold increase in risk and at times the blood pressure increases in hypertensive women. There is a 1.4–2.0 times increase in stroke, which increases with the age of contraceptive therapy.

There are major differences among the generations of oral contraceptive therapy. For example, a second generation OCP, levonorgesterol, is associated with an increased risk for myocardial infarction, whereas some of the fourth generation OCPs such as drospirenone do not increase but rather decrease blood pressure but nevertheless pose an increased risk for VTE. Thus the recommendation is for risk factor assessment and control in OCP users.^10, 11, 67–70^

#### Hormonal fertility therapy

The most comprehensive data on hormonal fertility therapy derive from a Canadian population cohort between 1993 and 2010. Among women who had successful fertility therapy, there was a decreased risk of all cause mortality, nonfatal coronary ischemia, stroke, TIA, thromboembolism, and heart failure that was present across all age and income groups, Obviously, this delineation likely reflects a healthy cohort selection bias.^71^

The women with unsuccessful fertility therapy had an increase in cardiovascular risk, with uncertainty as to whether this reflects different patient characteristics or possibly multiple cycles of hormonal therapies related to the lack of success of such therapy. Nonetheless, these women merit surveillance for subsequent cardiovascular events.^72^

#### Menopause and menopausal hormone therapy

Although coronary risk increases beginning about 10 years from the onset of menopause, premature menopause resulting from radiation, chemotherapy, or surgery increases the risk for coronary disease compared with women with natural menopause.

Despite this, the findings of the Heart and Estrogen Progestin Replacement Study (HERS) in women with coronary heart disease and the Women’s Health Initiative (in healthy women) provided conclusive evidence that menopausal hormone therapy is not beneficial for the prevention of cardiovascular disease,^73–75^ such that it is not recommended for cardiovascular primary or secondary prevention.

This is an area where clinical trial data dramatically altered both clinical recommendation and clinical practice. Based on clinical trials in women with coronary heart disease (HERS)^73^ and healthy women (Women’s Health Initiative),^74, 75^ menopausal hormone therapy is not recommended for the primary or secondary prevention of cardiovascular disease. Indeed, the USPSTF 2012 recommendations do not recommend menopausal hormone therapy for the primary prevention of any chronic condition.^76, 77^

### Polycystic ovary syndrome (PCOS)

PCOS is the most common endocrine disorder in women of reproductive age and places them at increased risk for insulin resistance, type 2 diabetes, and the development of the metabolic syndrome.^78–80^ PCOS women also have an increased prevalence of coronary calcium.^81^ Given their increased prevalence of cardiovascular risk factors, risk assessment and appropriate cardiovascular intervention is warranted.

#### Psychosocial factors (particularly depression)

More women than men have depression at all ages, with rates of depression in women nearly twice that for men.^82, 83^ The prevalence of depression is nearly 15% in a population of cardiac patients, 3 times that seen in the general population.^84^ In prospective studies, depression was associated with the development of CHD independent of other CHD risk factors.^85–87^ Some data supports correlation between the severity of depression and the risk of cardiovascular events.^88^ In women with symptoms of depression without known CVD, these symptoms were associated with a CHD risk in age-adjusted and multivariate models.^89^

Depressive symptoms occur in up to two-thirds of patients post-myocardial infarction,^90^ with major depressive disorder in almost 20%.^91, 92^

Anxiety has also been associated with an increased risk of fatal coronary heart disease in women.^93^

A recent statement from the American Heart Association suggests that healthcare provider consider the assessment and treatment of depression for its clinical benefits, although it has not been shown to improve cardiovascular outcomes.^94^

Psychosocial issues, particularly depression, preferentially disadvantage women. In the INTERHEART study, psychosocial factors were associated with greater cardiovascular mortality for women than men, 45.2% vs 28.8%. These factors included stress at work or at home; financial stress; and major life events. Depression in another database increased cardiovascular mortality 1.64-fold, independent of the severity of the depression. A component of this likely reflects both high-risk behaviors and non-adherence to therapy associated with depression. There is an increased mortality in depressed young women (younger than 55 years of age) with established coronary disease. Depression is also a risk factor for adverse outcomes in women with acute coronary syndromes. Likely there will be an increase in stress in this era of global violence and current global financial instability. Both in the U.S. and worldwide, a major issue involves cultural taboos in access to psychosocial care.^24, 95–98^

#### Aspirin for cardiovascular prevention

Aspirin is routinely recommended for the primary prevention of cardiovascular disease in men, but not in women.^99^ These latter recommendations derive from the Women’s Health Study^100^ which involved 38,876 healthy low-risk women older than age 45. Aspirin prevented stroke, but not myocardial infarction or cardiovascular death in women younger than 65 years of age, but with substantial potential for gastrointestinal bleeding. In women older than age 65 in the Women’s Health Study, there was a small percentage prevention of stroke, myocardial infarction, and cardiovascular death, but an almost equal increase in the risk of gastrointestinal bleeding, mandating individualization of the recommendations. These data are in sharp contrast to the Physicians’ Health Study, which involved only men. In men, there was a benefit of aspirin for myocardial infarction but not stroke. Of note is that the aspirin dosage in both the Women’s Health Study and the Physicians’ Health Study was 100 mg every other day, the standard regimen at the time these studies were initiated. Current recommendations are typically for 81 mg of aspirin daily, with larger doses associated with increased bleeding without increased benefit. The U.S. Preventive Services Task Force^101^ recommends low dose aspirin for CHD and CRC primary prevention for adults 50-59 years with a 10% or greater CVD risk not at increased risk of bleeding. Individualize at 60-69 years, but insufficient evidence for benefit:harm balance below 50 years or older than age 70. There are comparable gender recommendations for aspirin use for secondary prevention.

## References

[1] Mozaffarian D, Benjamin EJ, Go AS, Arnett DK, Blaha MJ, Cushman M, Das SR, de Ferranti S, Despres J-P, Fullerton HJ, Howard VJ, Huffman MD, Isasi CR, Jimenez MC, Judd SE, Kissela BM, Lichtman JH, Lisabeth LD, Liu S, Mackey RH, Magid DJ, McGuire DK, Mohler III ER, Moy CS, Muntner P, Mussolino ME, Nasir K, Neumar RW, Nichol G, Palaniappan L, Pandey DK, Reeves MJ, Rodriguez CJ, Rosamond W, Sorlie PD, Stein J, Towfighi A, Turan TN, Virani SS, Woo D, Yeh RW, Turner MB, on behalf of the American Heart Association Statistics Committee and Stroke Statistics Subcommittee (2015). Heart Disease and Stroke Statistics – 2016 Update. A report from the American Heart Association. Circulation.

[2] Wilson PWF, D’Agostino RB, Levy D, Belanger AM, Silbershatz H, Kannel WB (1998). Prediction of coronary heart disease using risk factor categories. Circulation.

[3] Lakoski SG, Greenland P, Wong ND, Schreiner PJ, Herrington DM, Kronmal RA, Liu K, Blumenthal RS (2007). Coronary artery calcium scores and risk for cardiovascular events in women classified as “low risk” based on Framingham Risk Score. The Multi-Ethnic Study of Atherosclerosis (MESA). Arch Intern Med.

[4] Ridker PM, Buring JE, Rifai N, Cook NR (2007). Development and validation of improved algorithms for assessment of global cardiovascular risk in women. The Reynolds Risk Score. JAMA.

[5] Mosca L, Benjamin EJ, Berra K, Bezanson JL, Dolor RJ, Lloyd-Jones DM, Newby LK, Pina IL, Roger VL, Shaw LJ, Zhao D, Beckie TM, Bushnell C, D’Armiento J, Kris-Etherton PM, Fang J, Ganiats TG, Gomes AS, Gracia CR, Haan CK, Jackson EA, Judelson DR, Kelepouris E, Lavie CJ, Moore A, Nussmeier NA, Ofili E, Oparil S, Ouyang P, Pinn VW, Sherif K, Smith Jr SC, Sopko G, Chandra-Strobos N, Urbina EM, Vaccarino V, Wenger NK (2011). Effectiveness-Based Guidelines for the Prevention of Cardiovascular Disease in Women – 2011 Update. A Guideline from the American Heart Association. Circulation.

[6] Goff DC, Lloyd-Jones DM, Bennett G, Coady S, D’Agostino RB, Gibbons R, Greenland P, Lackland DT, Levy D, O’Donnell CJ, Robinson JG, Schwartz JS, Shero ST, Smith Jr SC, Sorlie P, Stone NJ, Wilson PWF (2014). 2013 ACC/AHA Guideline for the Assessment of Cardiovascular Risk: A Report of the American College of Cardiology/American Heart Association Task Force on Practice Guidelines. Circulation.

[7] Rana JS, Tabada GH, Solomon MD, Lo JC, Jaffe MG, Sung SH, Ballantyne CM, Go AS (2016). Accuracy of the Atherosclerotic Cardiovascular Risk Equation in a large contemporary, multiethnic population. J Am Coll Cardiol.

[8] Cook NR, Ridker PM (2014). Further insight into the Cardiovascular Risk Calculator. The roles of statins, revascularizations, and underascertainment in the Women’s Health Study. JAMA Intern Med.

[9] DeFilippis AP, Young R, Carrubba CJ, McEnvoy JW, Budoff MJ, Blumenthal RS, Kronmal RA, McClelland RL, Nasir K, Blaha MJ (2015). An analysis of calibration and discrimination among multiple cardiovascular risk scores in a modern multiethnic cohort. Ann Intern Med.

[10] Bushnell C, McCullough LD, Awad IA, Chireau MV, Fedder WN, Furie KL, Howard VJ, Lichtman JH, Lisabeth LD, Pina IL, Reeves MJ, Rexrode KM, Saposnik G, Singh V, Towfighi A, Vaccarino V, Walters MR, on behalf of the American Heart Association Stroke Council, Council on Cardiovascular and Stroke Nursing, Council on Clinical Cardiology, Council on Epidemiology and Prevention, and Council for High Blood Pressure Research (2014). Guidelines for the prevention of stroke in women. A statement for healthcare professionals from the American Heart Association/American Stroke Association. Stroke.

[11] Bushnell C, McCullough L (2014). Stroke prevention in women: Synopsis of the 2014 American Heart Association/American Stroke Association Guideline. Ann Intern Med.

[12] Mieres JH, Gulati M, Bairey Merz N, Berman DS, Gerber TC, Hayes SN, Kramer CM, Min JK, Newby LK, Nixon JV (Ian), Srichai MB, Pellikka PA, Redberg RF, Wenger NK, Shaw LJ, on behalf of the American Heart Association Cardiac Imaging Committee of the Council on Clinical Cardiology and the Cardiovascular Imaging and Intervention Committee of the Council on Cardiovascular Radiology and Intervention (2014). Role of noninvasive testing in the clinical evaluation of women with suspected ischemic heart disease: A consensus statement from the American Heart Association. Circulation.

[13] Hlatky MA, Boineau RE, Higginbotham MB, Lee KL, Mark DB, Califf RM, Cobb FR, Pryor DB (1989). A brief self-administered questionnaire to determine functional capacity (the Duke Activity Status Index). Am J Cardiol.

[14] Mark DB, Shaw L, Harrell Jr FE, Hlatky MA, Lee KL, Bengtson JR, McCants CB, Califf RM, Pryor DB (1991). Prognostic value of a treadmill exercise score in outpatients with suspected coronary artery disease. N Engl J Med.

[15] Mark DB, Hlatky MA, Harrell Jr FE, Lee KL, Califf RM, Pryor DB (1987). Exercise treadmill score for predicting prognosis in coronary artery disease. Ann Intern Med.

[16] Alexander KP, Shaw LJ, Delong ER, Mark DB, Peterson ED (1998). Value of exercise treadmill testing in women. J Am Coll Cardiol.

[17] Sanfilippo AJ, Abdollah H, Knorr RX, Link C, Hopman W (2005). Stress echocardiography in the evaluation of women participating with chest pain syndrome: A randomized, prospective comparison with electrocardiographic stress testing. Can J Cardiol.

[18] Marwick TH, Anderson T, Williams MJ, Haluska B, Melin JA, Pashkow F, Thomas JD (1995). Exercise echocardiography is an accurate and cost-efficient technique for detection of coronary artery disease in women. J Am Coll Cardiol.

[19] Sawada SG, Ryan T, Fineberg NS, Armstrong WF, Judson WE, McHenry PL, Feigenbaum H (1989). Exercise echocardiographic detection of coronary artery disease in women. J Am Coll Cardiol.

[20] Williams MJ, Marwick TH, O’Gorman D, Foale RA (1994). Comparison of exercise echocardiography with an exercise score to diagnose coronary artery disease in women. Am J Cardiol.

[21] McSweeney JC, Rosenfeld AG, Abel WM, Braun LT, Burke LE, Daugherty SL, Fletcher GF, Gulati M, Mehta LS, Pettey C, Reckelhoff JF, on behalf of the American Heart Association Council on Cardiovascular and Stroke Nursing, Council on Clinical Cardiology, Council on Epidemiology and Prevention, Council on Hypertension, Council on Lifestyle and Cardiometabolic Health, and Council on Quality of Care and Outcomes Research (2016). Preventing and experiencing ischemic heart disease as a woman: State of the Science. A Scientific Statement from the American Heart Association. Circulation.

[22] Huxley R, Barzi F, Woodward M (2006). Excess risk of fatal coronary heart disease associated with diabetes in men and women: Meta-analysis of 37 prospective cohort studies. BMJ.

[23] Kalyani RR, Lazo M, Ouyang P, Turkbey E, Chevalier K, Brancati F, Becker D, Vaidya D (2014). Sex differences in diabetes and risk of incident coronary artery disease in healthy young and middle-aged adults. Diabetes Care.

[24] Yusuf S, Hawken S, Ounpuu S, Dans T, Avezum A, Lanas F, McQueen M, Budaj A, Pais P, Varigos J, Lisheng L, on behalf of the INTERHEART Study Investigators (2004). Effect of potentially modifiable risk factors associated with myocardial infarction in 52 countries (the INTERHEART study): case-control study. Lancet.

[25] Wannamethee SG, Papacosta O, Lawlor DA, Whincup PH, Lowe GD, Ebrahim S, Sattar N (2012). Do women exhibit greater differences in established and novel risk factors between diabetes and non-diabetes than men? The British Regional Heart Study and British Women’s Heart Health Study. Diabetologia.

[26] Peters SAE, Huxley RR, Woodward M (2014a). Diabetes as risk factor for incident coronary heart disease in women compared with men: A systematic review and meta-analysis of 64 cohorts including 858,507 individuals and 28,203 coronary events. Diabetologia.

[27] Peters SA, Huxley RR, Woodward M (2014b). Diabetes as a risk factor for stroke in women compared with men: A systematic review and meta-analysis of 64 cohorts, including 775,385 individuals and 12,539 strokes. Lancet.

[28] Huxley RR, Woodward M (2011). Cigarette smoking as a risk factor for coronary heart disease in women compared with men: A systematic review and meta-analysis of prospective cohort studies. Lancet.

[29] Willett WC, Green A, Stampfer MJ, Speizer FE, Colditz GA, Rosner B, Monson RR, Stason W, Hennekens CH (1987). Relative and absolute excess risk of coronary heart disease among women who smoke cigarettes. N Engl J Med.

[30] August P (2013). Hypertension in women. Adv Chronic Kidney Dis.

[31] Keyhani S, Scobie JV, Hebert PL, McLaughlin MA (2008). Gender disparities in blood pressure control and cardiovascular care in a national sample of ambulatory care visits. Hypertension.

[32] Go AS, Mozaffarian D, Roger VL, Benjamin EJ, Berry JD, Blaha MJ, Dai S, Ford ES, Fox CS, Franco S, Fullerton HJ, Gillespie C, Hailpern SM, Heit JA, Howard VJ, Huffman MD, Judd SE, Kissela BM, Kittner SJ, Lackland DT, Lichtman JH, Lisabeth LD, Mackey RH, Magid DJ, Marcus GM, Marelli A, Matchar DB, McGuire DK, Mohler III ER, Moy CS, Mussolino ME, Neumar RW, Nichol G, Pandey DK, Paynter NP, Reeves MJ, Sorlie PD, Stein J, Towfighi A, Turan TN, Virani SS, Wong ND, Woo D, Turner MB, on behalf of the American Heart Association Statistics Committee and Stroke Statistics Subcommittee (2013). Heart disease and stroke statistics – 2014 update. A report from the American Heart Association. Circulation.

[33] Chobanian AV, Bakris GL, Black HR, Cushman WC, Green LA, Izzo Jr JL, Jones DW, Materson BJ, Oparil S, Wright Jr JT, Roccella EJ, National High Blood Pressure Education Program Coordinating Committee (2003). The seventh report of the joint national committee on prevention, detection, evaluation, and treatment of high blood pressure. The JNC 7 report. JAMA.

[34] Hajjar I, Kotchen JM, Kotchen TA (2006). Hypertension: Trends in prevalence, incidence, and control. Ann Rev Public Health.

[35] Wolf HK, Tuomilehto J, Kuulasmaa K, Domarkiene S, Capaitis Z, Molarius A, Sans S, Dobson A, Keil U, Rywik S (1997). Blood pressure levels in the 41 populations of the WHO MONICA Project. J Hum Hypertens.

[36] Wenger NK, Lewis SJ, Welty FK, Herrington DM, Bittner V, on behalf of the TNT Steering Committee and Investigators (2008). Beneficial effects of aggressive low-density lipoprotein cholesterol lowering in women with stable coronary heart disease in the Treating to New Targets (TNT) study. Heart.

[37] Daly C, Clemens F, Lopez Sendon JL, Tavazzi L, Boersma E, Danchin N, Delahaye F, Gitt A, Julian D, Mulcahy D, Ruzyllo W, Thygesen K, Verheugt F, Fox KM, on behalf of the Euro Heart Survey Investigators (2006). Gender differences in the management and clinical outcome of stable angina. Circulation.

[38] Stone NJ, Robinson JG, Lichtenstein AH, Bairey Merz CN, Blum CB, Eckel RH, Goldberg AC, Gordon D, Levy D, Lloyd-Jones DM, McBride P, Schwartz JS, Shero ST, Smith Jr SC, Watson K, Wilson PWF (2014). 2013 ACC/AHA guideline on the treatment of blood cholesterol to reduce atherosclerotic cardiovascular risk in adults. A report of the American College of Cardiology/American Heart Association task force on practice guidelines. Circulation.

[39] Lloyd-Jones DM, Morris PB, Ballantyne CM, Birtcher KM, Daly Jr DD, DePalma SM, Minissian MB, Orringer MB, Orringer CE, Smith Jr SC (2016). 2016 ACC expert consensus decision pathway on the role of non-statin therapies for LDL-cholesterol lowering in the management of atherosclerotic cardiovascular disease risk. A report of the American College of Cardiology task force on clinical expert consensus documents. J Am Coll Cardiol.

[40] Eckel RH, Jakicic JM, Ard JD, de Jesus JM, Houston-Miller N, Hubbard VS, Lee I-M, Lichtenstein AH, Loria CM, Millen BE, Nonas CA, Sacks FM, Smith Jr SC, Svetkey LP, Wadden TA, Yanovski SZ (2014). 2013 AHA/ACC guideline on lifestyle management to reduce cardiovascular risk. A report of the American College of Cardiology/American Heart Association task force on practice guidelines. Circulation.

[41] Kostis WJ, Cheng JQ, Dobrzynski JM, Cabrera J, Kostis JB (2012). Meta-analysis of statin effects in women versus men. J Am Coll Cardiol.

[42] Jensen MD, Ryan DH, Apovian CM, Ard JD, Comuzzie AG, Donato KA, Hu FB, Hubbard VS, Jakicic JM, Kushner RF, Loria CM, Millen BE, Nonas CA, Pi-Sunyer FX, Stevens J, Stevens VJ, Wadden TA, Wolfe BM, Yanovski SZ (2014). 2013 AHA/ACC/TOS guideline for the management of overweight and obesity in adults: A report of the American College of Cardiology/American Heart Association task force on practice guidelines and the obesity society. Circulation.

[43] Global Status Report on Noncommunicable Disease (2010). World Health Organization, Geneva, 2011.

[44] Manson JE, Rimm EB, Stampfer MJ, Colditz GA, Willett WC, Krolewski AS, Rosner B, Hennekens CH, Speizer FE (1991). Physical activity and incidence of non-insulin-dependent diabetes mellitus in women. Lancet.

[45] Hu FB, Stampfer MJ, Solomon C, Liu S, Colditz GA, Speizer FE, Willett WC, Manson JE (2001). Physical activity and risk for cardiovascular events in diabetic women. Ann Intern Med.

[46] Roman MJ, Shanker B-A, Davis A, Lockshin MD, Sammaritano L, Simantov R, Crow MK, Schwartz JE, Paget SA, Devereux RB, Salmon JE (2003). Prevalence and correlates of accelerated atherosclerosis in systemic lupus erythematosus. N Engl J Med.

[47] del Rincon ID, Williams K, Stern MP, Freeman GL, Escalante A (2001). High incidence of cardiovascular events in a rheumatoid arthritis cohort not explained by traditional cardiac risk factors. Arthritis Rheum.

[48] Solomon DH, Karlson EW, Rimm EB, Cannuscio CC, Mandl LA, Manson JE, Stampfer MJ, Curhan GC (2003). Cardiovascular morbidity and mortality in women diagnosed with rheumatoid arthritis. Circulation.

[49] Avina-Zubieta JA, Choi HK, Sadatsafavi M, Etminan M, Esdaile JM, Lacaille D (2008). Risk of cardiovascular mortality in patients with rheumatoid arthritis: A meta-analysis of observational studies. Arthritis Rheum.

[50] Asanuma Y, Oeser A, Shintani AK, Turner E, Olsen N, Fazio S, Linton MF, Raggi P, Stein CM (2003). Premature coronary-artery atherosclerosis in systemic lupus erythematosus. N Engl J Med.

[51] Kahlenberg JM, Kaplan MJ (2013). Mechanisms of premature atherosclerosis in rheumatoid arthritis and lupus. Annu Rev Med.

[52] Urowitz MB, Gladman DD, Anderson NM, Su J, Romero-Diaz J, Bae SC, Fortin PR, Sanchez-Guerrero J, Clarke A, Bernatsky S, Gordon C, Hanly JG, Wallace DJ, Isenberg D, Rahman A, Merrill J, Ginzler E, Alarcon GS, Fessler BF, Petri M, Bruce IN, Kamashta M, Aranow C, Dooley M, Manzi S, Ramsey-Goldman R, Sturfelt G, Nived O, Steinsson K, Zoma A, Ruiz-Irastorza G, Lim S, Kalunian KC, Inanc M, van Vollenhoven R, Ramos-Casals M, Kamen DL, Jacobsen S, Peschken C, Askanase A, Stoll T (2016). Cardiovascular events prior to or early after diagnosis of systemic lupus erythematosus in the systemic lupus international collaborating clinics cohort. Lupus Sci Med.

[53] Zhang J, Chen L, Delzell E, Munter P, Hillegass WB, Safford MM, Millan IYN, Crowson CS, Curtis JR (2014). The association between inflammatory markers, serum lipids and the risk of cardiovascular events in patients with rheumatoid arthritis. Ann Rheum Dis.

[54] Horreau C, Pouplard C, Brenaut E, Barnetche T, Misery L, Cribier B, Jullen D, Aractingi S, Aubin F, Joly P, Le Maitre M, Ortonne J-P, Paul C, Richard M-A (2013). Cardiovascular morbidity and mortality in psoriasis and psoriatic arthritis: A systemic literature review. JEADV.

[55] Salmon JE, Roman MJ (2008). Subclinical atherosclerosis in rheumatoid arthritis and systemic lupus erythematosus. Am J Med.

[56] Nardi O, Zureik M, Courbon D, Ducimetiere P, Clavel-Chapelon F (2006). Preterm delivery of a first child and subsequent mothers’ risk of ischaemic heart disease: A nested case-control study. Eur J Cardiovasc Prev Rehabil.

[57] Catov JM, Wu CS, Olsen J, Sutton-Tyrrell K, Li J, Nohr EA (2010). Early or recurrent preterm birth and maternal cardiovascular disease risk. Ann Epidemiol.

[58] Bukowski R, Davis KE, Wilson PWF (2012). Delivery of a small for gestational age infant and greater maternal risk of ischemic heart disease. PLOS ONE.

[59] Bellamy L, Casas J-P, Hingorani AD, Williams DJ (2007). Pre-eclampsia and risk of cardiovascular disease and cancer in later life: Systematic review and meta-analysis. BMJ.

[60] Ahmed R, Dunford J, Mehran R, Robson S, Kunadian V (2014). Pre-eclampsia and future cardiovascular risk among women. J Am Coll Cardiol.

[61] Wenger NK (2014). Recognizing pregnancy-associated cardiovascular risk factors. Am J Cardiol.

[62] Fraser A, Nelson SM, Macdonald-Wallis C, Cherry L, Butler E, Sattar N, Lawlor DA (2012). Associations of pregnancy complications with calculated cardiovascular disease risk and cardiovascular risk factors in middle age. The Avon Longitudinal Study of Parents and Children. Circulation.

[63] Bellamy L, Casas J-P, Hingorani AD, Williams D (2009). Type 2 diabetes mellitus after gestational diabetes: A systematic review and meta-analysis. Lancet.

[64] Carr DB, Utzschneider KM, Hull RL, Tong J, Wallace TM, Kodama K, Shofer JB, Heckbert SR, Boyko EJ, Fujimoto WY, Kahn SE, American Diabetes Association GENNID Study Group (2006). Gestational diabetes mellitus increases the risk of cardiovascular disease in women with a family history of type 2 diabetes. Diabetes Care.

[65] Bentley-Lewis R (2009). Late cardiovascular consequences of gestational diabetes mellitus. Semin Reprod Med.

[66] Pemu PI, Ofili E (2008). Hypertension in women –Part II. J Clin Hypertens.

[67] Poulter NR, Chang CL, Farley TMM, Meirik O, Marmot MG (1996). WHO Collaborative Study of Cardiovascular Disease and Steroid Hormone Contraception. Ischaemic stroke and combined oral contraceptives: Results of an international, multicenter, case-control study. Lancet.

[68] Lubianca JN, Faccin CS, Fuchs FD (2003). Oral contraceptives: A risk factor for uncontrolled blood pressure among hypertensive women. Contraception.

[69] Shufelt CL, Bairey Merz CN (2009). Contraceptive hormone use and cardiovascular disease. J Am Coll Cardiol.

[70] Tanis BC, van den Bosch MAAJ, Kemmeren JM, Cats VM, Helmerhorst FM, Algra A, van der Graaf Y, Rosendaal FR (2001). Oral contraceptives and the risk of myocardial infarction. N Engl J Med.

[71] Udell JA, Lu H, Redelmeier DA (2013). Long-term cardiovascular risk in women prescribed fertility therapy. J Am Coll Cardiol.

[72] Udell JA, Lu H, Redelmeier DA (2017). Failure of fertility therapy and subsequent adverse cardiovascular events. CMAJ.

[73] Hulley S, Grady D, Bush T, Furberg C, Herrington D, Riggs B, Vittinghoff E, for the Heart and Estrogen/progestin Replacement Study (HERS) Research Group (1998). Randomized trial of estrogen plus progestin for secondary prevention of coronary heart disease in postmenopausal women. JAMA.

[74] Rossouw JE, Anderson GL, Prentis RL, LcCroix AZ, Kooperberg C, Stefanick ML, Jackson RD, Beresford SAA, Howard BV, Johnson KC, Kotchen JM, Ockene J, Writing Group for the Women’s Health Initiative Investigators (2002). Risks and benefits of estrogen plus progestin in healthy postmenopausal women. Principal results from the Women’s Health Initiative Randomized Controlled Trial. JAMA.

[75] Anderson GL, Limacher M, Assaf AR, Bassford T, Beresford SAA, Black H, Bonds D, Brunner R, Brzyski R, Caan B, Chlebowski R, Curb D, Gass M, Hays J, Heiss G, Hendrix S, Howard BV, Hsia J, Hubbell A, Jackson R, Johnson KC, Judd H, Kotchen JM, Kuller L, LaCroix AZ, Lane D, Langer RD, Lasser N, Lewis CE, Manson J, Margolik Ockene J, O’Sullivan MJ, Phillips L, Prentice RL, Ritenbaugh C, Robbins J, Rossouw JE, Sarto G, Stefanik ML, Horn L, Wactawski-Wende J, Wallace R, Wassertheil-Smoller S, The Women’s Health Initiative Steeering Committee (2004). Effects of conjugated equine estrogen in postmenopausal women with hysterectomy. The Women’s Health Initiative Randomized Controlled Trial. JAMA.

[76] Moyer VA, on behalf of the U.S (2013). Preventive Services Task. Force Menopausal hormone therapy for the primary prevention of chronic conditions: U.S. Preventive Services Task Force Recommendation Statement. Ann Intern Med.

[77] Nelson HD, Walker M, Zakher B, Mitchell J (2012). Menopausal hormone therapy for the primary prevention of chronic conditions: A systematic review to update the U.S. Preventive Services Task Force. Recommendations. Ann Intern Med.

[78] Dokras A, Bochner M, Hollinrake E, Markham S, van Voorhis B, Jagasia DH (2005). Screening women with polycystic ovary syndrome for metabolic syndrome. Obstet Gynecol.

[79] Dokras A (2008). Cardiovascular disease risk factors in polycystic ovary syndrome. Semin Reprod Med.

[80] Ehrmann DA, Liljenquist DR, Kasza K, Azziz R, Legro RS, Ghazzi MN, for the PCOS/Troglitazone Study Group (2006). Prevalence and predictors of the metabolic syndrome in women with polycystic ovary syndrome. J Clin Endrocrinol Metab.

[81] Christian RC, Dumesic DA, Behrenbeck T, Oberg AL, Sheedy PF II, Fitzpatrick LA (2003). Prevalence and predictors of coronary artery calcification in women with polycystic ovary syndrome. J Clin Endocrinol Metab.

[82] Kessler RC, Berglund P, Demler O, Jin R, Koretz D, Merikangas KR, Rush AJ, Walters EE, Wang PS (2003). The epidemiology of major depressive disorder. Results from the National Comorbidity Survey Replication (NCS-R). JAMA.

[83] Kessler RC (2003). Epidemiology of women and depression. J Affect Disord.

[84] Colquhoun DM, Bunker SJ, Clarke DM, Glozier N, Hare DL, Hickie IB, Tatoulis J, Thompson DR, Tofler GH, Wilson A, Branagan MG (2013). Screening, referral and treatment for depression in patients with coronary heart disease. A consensus statement from the National Heart Foundation of Australia. Med J Aust.

[85] Wulsin LR, Singal BM (2003). Do depressive symptoms increase the risk for the onset of coronary disease: A systematic quantitative review. Psychosom Med.

[86] Anda R, Williamson D, Jones D, Macera C, Eaker E, Glassman A, Marks J (1993). Depressed affect, hopelessness, and the risk of ischemic heart disease in a cohort of U.S. adults. Epidemiology.

[87] Ariyo AA, Haan M, Tangen CM, Rutledge JC, Cushman M, Dobs A, Furberg CD, for the Cardiovascular Health Study Collaborative Research Group (2000). Depressive symptoms and risks of coronary heart disease and mortality in elderly Americans. Circulation.

[88] Lesperance F, Frasure-Smith N, Talajic M, Bourassa MG (2002). Five-year risk of cardiac mortality in relation to initial severity and one-year changes in depression symptoms after myocardial infarction. Circulation.

[89] Whang W, Kubzansky LD, Kawachi I, Rexrode KM, Kroenke CH, Glynn RJ, Garan H, Albert CM (2009). Depression and risk of sudden cardiac death and coronary heart disease in women. Results from the Nurses’ Health Study. J Am Coll Cardiol.

[90] Cay EL, Vetter N, Philip AE, Dugard P (1972). Psychological status during recovery from an acute heart attack. J Psychosom Res.

[91] Thombs BD, Bass EB, Ford DE, Stewart KJ, Tsilidis KK, Patel U, Fauerbach JA, Bush DE, Ziegelstein RC (2006). Prevalence of depression in survivors of acute myocardial infarction. Review of the evidence. J Gen Intern Med.

[92] Carney RM, Freedland KE (2003). Depression, mortality, and medical morbidity in patients with coronary heart disease. Biol Psychiatry.

[93] Albert CM, Chae CU, Rexrode KM, Manson JE, Kawachi I (2005). Phobic anxiety and risk of coronary heart disease and sudden cardiac death among women. Circulation.

[94] Smith Jr SC, Benjamin EJ, Bonow RO, Braun LT, Creager MA, Franklin BA, Gibbons RJ, Grundy SM, Hiratzka LF, Jones DW, Lloyd-Jones DM, Minissian M, Mosca L, Peterson ED, Sacco RL, Spertus J, Stein JH, Taubert KA (2011). AHA/ACCF secondary prevention and risk reduction therapy for patients with coronary and other atherosclerotic vascular disease: 2011 update. A guideline from the American Heart Association and American College of Cardiology Foundation. Circulation.

[95] Shah AJ, Ghasemzadeh N, Zaragoza-Macias E, Patel R, Eapen DJ, Neeland IJ, Piple PM, Zafari AM, Quyyumi AA, Vaccarino V (2014). Sex and age differences in the association of depression with obstructive coronary artery disease and adverse cardiovascular events. J Am Heart Assoc.

[96] Litchman JH, Froelicher ES, Blumenthal JA, Carney RM, Doering LV, Frasure-Smith N, Freedland KE, Jaffe AS, Leifheit-Limson EC, Sheps DS, Vaccarino V, Wulsin L, on behalf of the American Heart Association Statistics Committee of the Council on Epidemiology and Prevention and the Council on Cardiovascular and Stroke Nursing (2014). Depression as a risk factor for poor prognosis among patients with acute coronary syndrome: Systematic review and recommendations. A scientific statement from the American Heart Association. Circulation.

[97] Rosengren A, Hawken S, Ounpuu S, Sliwa K. Zubaid M, Almahmeed WA, Blackett KN, Sitthi-amorn C, Sato H, Yusuf S, for the INTERHEART Investigators (2004). Association of psychosocial risk factors with risk of acute myocardial infarction in 11,119 cases and 13,648 controls from 52 countries (the INTERHEART study): Case-control study. Lancet.

[98] Rutledge T, Linke SE, Johnson BD, Bittner V, Krantz DS, Cornell CE, Vaccarino V, Pepine CJ, Handberg EM, Eteiba W, Shaw LJ, Parashar S, Eastwood J-A, Vido DA, Bairey Merz CN (2012). Relationship between cardiovascular disease risk factors and depressive symptoms as predictors of cardiovascular disease events in women. J Women’s Health.

[99] Berger JS, Roncaglioni MC, Avanzini F, Pangrazzi I, Tognoni G, Brown DL (2006). Aspirin for the primary prevention of cardiovascular events in women and men. A sex-specific meta-analysis of randomized controlled trials. JAMA.

[100] Ridker PM, Cook NR, Lee I-M, Gordon D, Gaziano JM, Manson JE, Hennekens CH, Buring JE (2005). A randomized trial of low-dose aspirin in the primary prevention of cardiovascular disease in women. N Engl J Med.

[101] Bibbins-Domingo K, on behalf of the U.S. Preventive Services Task Force (2016). Aspirin use for the primary prevention of cardiovascular disease and colorectal cancer: U.S. Preventive Services Task Force Recommendation Statement. Ann Intern Med.

